# Binge eating disorder and coronavirus outbreak among health care workers in tunisia

**DOI:** 10.1192/j.eurpsy.2021.777

**Published:** 2021-08-13

**Authors:** M. Dhemaid, W. Abbes, M. Tfifha, K. Medhaffer, M. Abbes, L. Ghanmi

**Affiliations:** Psychiatry, regional hospital of Gabes, Gabes, Tunisia

**Keywords:** mental health, Binge eating, COVID-19, health care workers

## Abstract

**Introduction:**

COVID19 outbreak had affected physical and mental health of individuals. Different adverse health behaviors had worsened and eating disorders had evolved. Health care workers were not spared.

**Objectives:**

To screen binge eating disorder among health care workers of regional hospital of Gabes (south of Tunisia) and its associated factors.

**Methods:**

We conducted a cross-sectional, descriptive and analytical study, from April 19, 2020, to May 5, 2020 on 289 in Gabes regional Hospital. All healthcare workers were included (n=620). Workers who were on sick leave during the study were excluded. During this period, the total confirmed cases of COVID-19 exceeded 900 cases in Tunisia and around 20 cases in Gabes. We used a self-administered anonymous questionnaire containing sociodemographic and clinical data. DSM-5 diagnostic criteria were used to assess Binge-Eating Disorder.

**Results:**

Of the 289 responding participants, 85 were physicians (29%), 166 nurses (57.4%), 8 ambulance drivers (2.8%) and 30 health-related administrators (10.3%). A total of 100 participants (34.6%) were frontline health care workers directly engaged in diagnosing, treating or caring for patients with coronavirus disease. Nine percent of participants experienced binge eating disorder during the outbreak. Binge eating disorders were associated to past psychiatric history of eating disorder (p=0.001), social isolation (p=0.001), increased consumption of tea and coffee (p=0.02) and the fact of being a frontline care giver (p=0.009).

**Conclusions:**

Binge eating disorders are usually associated with health problems: obesity and consequently severe form of coronavirus disease. Screening those disorders is important to alleviate its physical impact.

